# Exosomal microRNAs as Potential Biomarkers and Therapeutic Agents for Acute Ischemic Stroke: New Expectations

**DOI:** 10.3389/fneur.2021.747380

**Published:** 2022-01-24

**Authors:** Yingzhi Xu, Yue Hu, Shixin Xu, Fengzhi Liu, Ying Gao

**Affiliations:** ^1^Institute for Brain Disorders, Beijing University of Chinese Medicine, Beijing, China; ^2^Department of Neurology, Dongzhimen Hospital, Beijing University of Chinese Medicine, Beijing, China; ^3^Key Laboratory of Chinese Internal Medicine of Ministry of Education and Beijing, Dongzhimen Hospital, Beijing University of Chinese Medicine, Beijing, China; ^4^School of Integrated Chinese and Western Medicine, Nanjing University of Chinese Medicine, Nanjing, China; ^5^Medical Experiment Center, First Teaching Hospital of Tianjin University of Traditional Chinese Medicine, Tianjin, China; ^6^Tianjin Key Laboratory of Translational Research of TCM Prescription and Syndrome, Tianjin, China

**Keywords:** exosomal miRNAs, acute ischemic stroke, diagnosis, treatment, mechanism

## Abstract

The morbidity and mortality rates of ischemic stroke (IS) are very high, and IS constitutes one of the main causes of disability and death worldwide. The pathogenesis of ischemic stroke includes excitotoxicity, calcium overload, oxygen radical injury, inflammatory reactions, necrosis/apoptosis, destruction of the blood-brain barrier (BBB), and other pathologic processes. Recent studies have shown that exosomes are critical to the pathogenesis, diagnosis, and treatment of cerebral infarctions resulting from ischemic stroke; and there is growing interest in the role of exosomes and exosomal miRNAs in the diagnosis and treatment of IS. Exosomes from central nervous system cells can be found in cerebrospinal fluid and peripheral bodily fluids, and exosomal contents have been reported to change with disease occurrence. Exosomes are small membranous extracellular vesicles (EVs), 30–150 nm in diameter, that are released from the cell membrane into the depressions that arise from the membranes of multivesicular bodies. Exosomes carry lipids, proteins, mRNAs, and microRNAs (miRNAs) and transport information to target cells. This exosomal transfer of functional mRNAs/miRNAs and proteins ultimately affects transcription and translation within recipient cells. Exosomes are EVs with a double-membrane structure that protects them from ribonucleases in the blood, allowing exosomal miRNAs to be more stable and to avoid degradation. New evidence shows that exosomes derived from neural cells, endothelial cells, and various stem cells create a fertile environment that supports the proliferation and growth of neural cells and endothelial cells, inhibits apoptosis and inflammatory responses, and promotes angiogenesis. In the present review, we discuss how circulating exosomes—and exosomal miRNAs in particular—may provide novel strategies for the early diagnosis and treatment of ischemic stroke via their potential as non-invasive biomarkers and drug carriers.

## Introduction

Ischemic stroke (IS) is one of the major causes of disability and mortality worldwide, and exhibits high rates of incidence and recurrence. In 2019, stroke was ranked second of the 10 leading causes of disability-adjusted life years (DALYs) for both individuals 50–74 years of age and those ≥ 75 years of age (1, 2). IS exhibits high rates of mortality and physical disability, and has become a heavy burden for individuals and society, especially in low-income and middle-income countries. The principal type of stroke is IS, which accounts for 84.4%; primary hemorrhagic stroke accounts for the majority of the remainder (1). Cerebral ischemia can lead to a series of pathologic changes that ultimately lead to irreparable neuronal damage. The accompanying pathogenesis includes excitotoxicity, calcium overload, oxygen-radical injury, inflammatory reactions, necrosis/apoptosis, destruction of the blood-brain barrier (BBB), and other pathologic processes.

Due to the scarcity of treatment methods and the thrombolytic time-window, the thrombolysis rate in stroke patients is very low (3). Thus, clinical diagnosis of IS now primarily depends on magnetic resonance imaging and computed tomography; however, due to a patient's physical manifestations, such as the placement of a metal stent or steel plate implantation, it is not reasonable to accept MRI as the sole modality. In addition, most community medical institutions lack the appropriate professional technicians and testing equipment, which limits the clinical application of MRI. Therefore, an optimized and clinically operable biomarker is needed for the early and accurate diagnosis of IS. Importantly, recent studies have shown that exosomes occupy a significant position in the pathogenesis, diagnosis, and treatment of cerebral infarction. Some nerve cells can synthesize and release exosomes after stroke, which then pass through the BBB. For example, exosomes released from brain cells are detectable in peripheral blood and/or cerebrospinal fluid (CSF) (4–6). In addition, endothelial and blood cells release exosomes into the blood after a stroke, while nerve cells release exosomes into the cerebrospinal fluid; with the latter distributed within and outside the brain through the BBB (7). These exosomes may therefore be useful as biomarkers that reflect stroke-induced pathologic processes, and might have potential as drug carriers for promoting recovery.

An exosome is a small membranous extracellular vesicle (EV) produced by the membrane of multivesicular bodies (MVBs), and when the endosomes or MVBs fuse with the plasma membrane, exosomes are released extracellularly (8). Exosomes have a diameter of 30–150 nm (9, 10), and transfer lipids, proteins, mRNAs, and microRNAs (miRNAs) (11). Furthermore, they play an important role in intercellular communication, maintenance of myelin sheaths, and the elimination of cellular waste (12, 13). Exosomes can cross the BBB and possess a double- membrane structure that protects them from ribonucleases in blood, making exosomal miRNAs highly stable and preventing them from undergoing degradation (14–16). Thus, exosomal miRNAs may represent an ideal biomarker for circulating bodily fluids. As exosomal contents also change commensurately with disease development, neurocyte-derived exosomes that cross the BBB can be used as valuable biomarkers of nervous system diseases (17, 18). Exosomes from miRNA-overexpressing cells—e.g., stem cells and other cultured cells—have been shown to protect against ischemia-induced neural injury, the underlying mechanisms of which are related to inhibiting inflammatory reactions, promoting angiogenesis, regulating autophagy, and promoting neural repair. In this review, we principally discuss the most recent understanding of exosomes and exosomal miRNAs, and introduce the potential of using exosomal miRNAs in treating ischemic diseases. We also examine the mechanisms involving exosomes in these processes and review the research on circulating exosomal miRNAs as potential diagnostic biomarkers for stroke at different stages. Finally, we highlight important characteristics of exosomes and exosomal miRNAs in the potential diagnosis and treatment of IS so as to better understand the potential governing mechanisms and provide more-effective strategies for the use of exosomal miRNA in the diagnosis and treatment of IS.

## Exosomes and Exosomal miRNAs

### Exosome Formation

**Figure 1:**
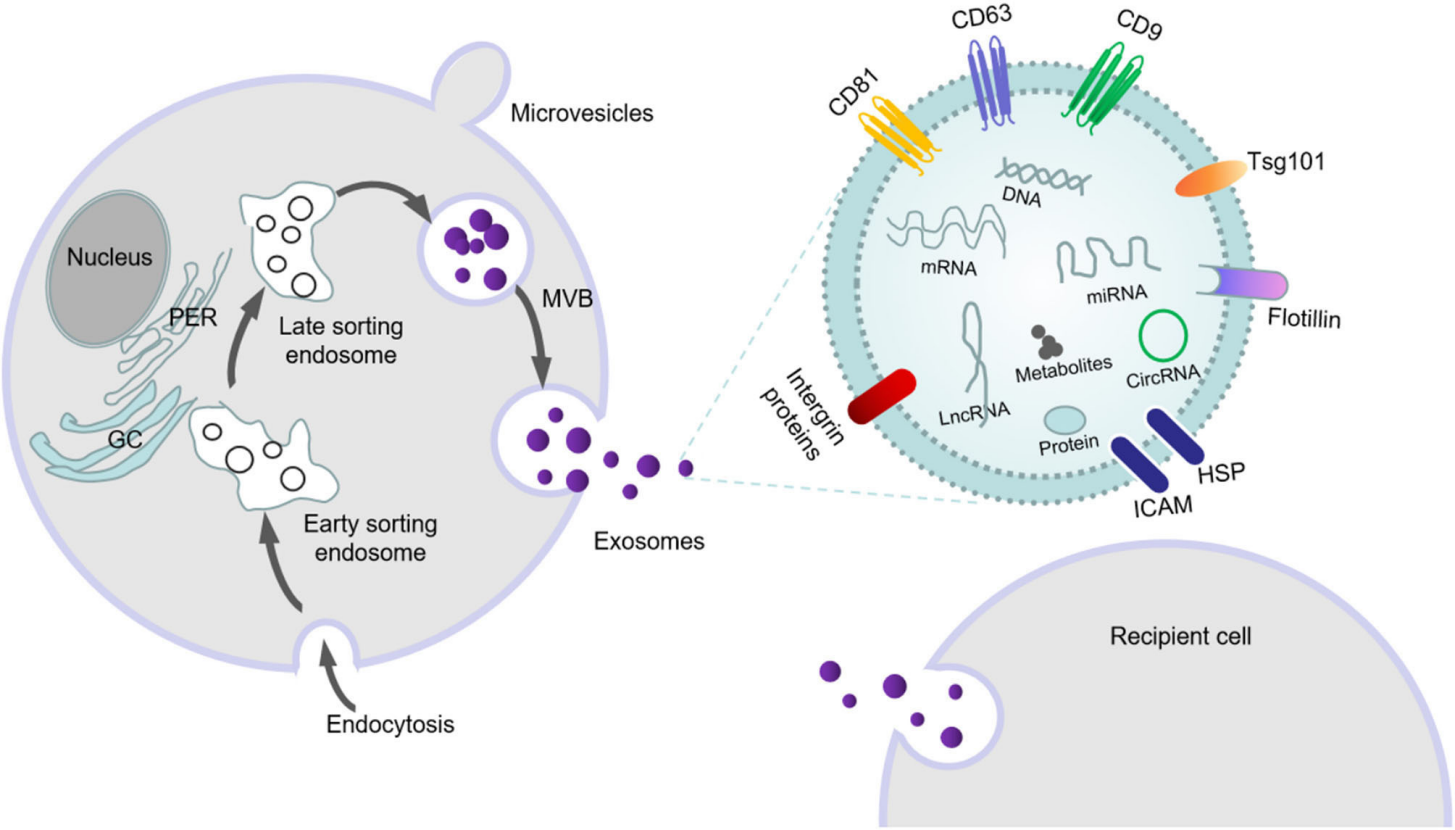
The formation, secretion and swallowing of exosomes, as well as the substances carried by exosomes.

Exosomal formation consists of the following stages. Extracellular components (e.g., proteins, lipids, RNAs, miRNAs, metabolites) and cell membrane-surface proteins migrate inward into the cell after the plasmalemma invaginates and form early-sorting endosomes (ESEs). After maturation, intraluminal vesicles (ILVs) begin to form within the lumens of these vesicles by wrapping the limiting membrane. Then, these ILVs isolate cytoplasmic molecules, which leads to their accumulation in late endosomes and the formation of MVBs (19). Thereafter, MVBs fuse with the lysosome and plasma membrane; and at the lysosomal and plasma membranes, MVBs release their contents out of the cell (Figure 1).

This process is primarily accomplished by the ESCRT system, which is mainly composed of four complexes—ESCRT-0, ESCRT-I, ESCRT-II, and ESCRT-III—and accessory proteins (VPS4, VTA1 and ALIX) that are involved in intraluminal vesicle formation and cargo sorting (20–22). ESCRT-0 binds to specific receptors at the endosomal membrane and is then transferred to ESCRT-I and ESCRT-II. The two proteins can transform the membrane into buds with barriers, and then activate ESCRT-III and VPS4 ATPase to drive vesicle rupture to form intracavitary vesicles (23). By identifying and aggregating substrates, an internal vesicle (i.e., intraluminal vesicle) is formed by wrapping it in bud ILVs. ESCRT-III identifies internal vesicles by Alix proteins and forms a late mature inclusion body, called the polycystic corpuscle (24). The formation of MVBs can also be mediated through non-ESCRT-dependent sorting mechanisms such as ceramide-like sphingolipid-signaling molecules (25), four-step transmembrane glycoproteins (26, 27), and Rab GTP enzymes (28). Previous studies have shown that Rab family small GTPases (Rab5, Rab7, Rab27A, and Rab27b) are involved in the transport of multivesicular endosomes and their fusion with the plasma membrane and/or lysosomes (29, 30). A recent study has shown that Rab31 marks and controls an ESCRT-independent exosomal pathway. Active Rab31 is phosphorylated by epidermal growth factor receptor (EGFR) and binds to flotillin protein in lipid-raft microdomains, promoting the entrance of EGFR into MVEs and forming ILVs; RAB31protein can also suppress MVE degradation. Rab31 recruits the GTPase-activating protein TBC1D2B to inactivate Rab7, which prevents MVE from fusing with lysosomes and favors the secretion of ILVs as exosomes (31). When MVBs fuse with the cytoplasmic membrane, they release vesicles into the extracellular space by exocytosis via the formation of an exocrine body. Exocrine bodies can use autocrine-, paracrine-, or hormone-like secretion to enable their encapsulated signaling molecules to interact with and modulate recipient cells (24).

### Biologic Characteristics of Exosomes

Exosomes are the most widely studied class of EVs released by mammalian cells, and exocrine products are released by cells in all organisms. EVs can be divided into the following three types according to their diameter: microparticles/microvesicles (100–1,000 nm); exosomes (30–150 nm); and apoptotic bodies (500–4,000 nm) (9, 32, 33). The size and composition of apoptotic bodies are highly heterogeneous, and they are formed in the cytoplasmic membrane at the last stage of apoptosis. In contrast, exosomes are endocytic, and microbubbles are formed by directly sprouting from the plasma membrane. Exosomes are the smallest EVs, arise from MVB membranes (34, 35), and are cup-shaped under electron microscopy (36); compared with other types of EVs, their characteristics are more uniform. Exosomes form intraluminal vesicles through a process that involves endocytosis; and when the endosomes or MVB fuse with the plasma membrane at the late stage, the exosomes are released into the extracellular space on the plasma membrane (24, 37). After ultracentrifugation (100,000 g), the relatively pure exosomes are separated by a sucrose gradient. Circulating exosomes that have been isolated have diameters between 30 and 100 nm. Exosomes can be identified by specific exosomal protein markers: members of the tetrad family cluster of differentiation 9 (CD9), cluster of differentiation 63 (CD63), cluster of differentiation 81 (CD81), and tumor-susceptibility gene 101 (Tsg101) (38, 39) (Figure 1).

Exosomes can be isolated from many types of bodily fluids—including cerebrospinal fluid, serum, plasma, urine, saliva, breast milk, semen, ascites, and amniotic fluid. In addition to lipids and proteins, exosomes also contain mRNAs and miRNAs from the host cell (40). The ExoCarta database (www.exocarta.org) lists 9,769 proteins, 3,408 mRNAs, 2,838 miRNAs, and 1,116 lipids (41) as candidate exosomal cargos (42); however, exosomes secreted from different cell lines contain different substances. Since the first report of exosomes in 2007, many investigators have reported findings on exosomal miRNAs. In the CNS, many types of cells—including neurons, microglia, astrocytes, and oligodendrocytes—can secrete exosomes (43). Additionally, exosomal miRNAs have been shown to play a significant role in intercellular communication within the CNS, as well as in the pathogenesis and treatment of neurological diseases. However, the mechanisms by which exosomes selectively carry their contents remain unclear. While there are reports that specific sequences are needed to classify mRNAs or miRNAs in exosomes (44, 45), reliable criteria for the selection of lipids, proteins, and miRNAs into exosomes continue to be arcane.

### Exosomal miRNAs

Exosomes contain miRNAs, proteins, mRNAs, long non-coding RNAs (lncRNAs), lipids, and other signaling substances; and act on adjacent or distal cells through autocrine and paracrine targeting to carry out information exchange between cells (46, 47). The lipid-bilayer structure of exosomes keeps cargo miRNAs stable from enzymatic dissolution in bodily fluids. Thus, exosomes are carriers of miRNAs, and miRNAs can in this way successfully avoid immune rejection, and endocytose into target cells and be delivered to these cells (48). MiRNAs in exosomes not only function in recipient cells, but can also carry genetic information to communicate between cells, thus affecting biologic behavior and pathophysiologic development of cells. MiRNAs are critically involved in many biologic activities, including cellular genesis, differentiation, and signal transduction (49–52). In addition, miRNAs are key regulators of gene-expression networks, which can induce cellular proliferation, death, and migration—as well as enhance immune responses and induce angiogenesis (53–56). The miRNA database, miRBase, currently contains information on more than 2,500 human miRNAs (57). After detection, there are more than 45,000 conserved target gene sites in the human genome, and over 60% of coding genes can be combined with miRNAs—which is enough to account for the wide range of regulatory functions by miRNAs (57, 58). MiRNAs may additionally serve as non-invasive biomarkers for many disease states such as tumors, diabetes, and lipid disorders (59, 60). Exosomal miRNAs participate in a variety of biologic processes such as inflammation, cellular proliferation, differentiation, and apoptosis, and thus may regulate a variety of pathologic processes. Studies have found that exosomal miRNAs are important in the pathogenesis of stroke, dementia, Parkinson's disease, and other CNS diseases (61–64). Furthermore, they can pass through the BBB, with the accompanying characteristics of non-immunogenicity and rapid extraction. Therefore, exosomal miRNAs are promising as new biomarkers for the diagnosis and treatment strategy of neurologic diseases (65–67).

As one of the important components of secreted body weight, how miRNAs are loaded into exosomes is not completely clear. The possible loading modes reported at present include the following three pathways. (1) The neutral sphingomyelinase 2 (n smase2)-dependent pathway: n smase2 can increase the amount/number of secreted miRNA. When the expression and function of n smase2 are specifically inhibited, the number of secreted miRNAs decreases, indicating that n smase2 is involved in the packaging of miRNA into exosomes (68). (2) The heterogeneous ribonucleoprotein (HN RNP)-dependent pathway: the ggag motif of the 3 ‘part of the miRNA sequence can be recognized by HN RNP. Once this recognition occurs, specific miRNAs are then packaged into exosomes (69). (3) The miRNA-induced silencing complex (mirisc)-related pathway: when the main component of mirisc-ago2 was knocked out, the content of miRNAs transported in specific HEK293T cell exosomes diminished (70). Some studies have also shown that MI RISC is related to the sorting mechanism of secreted MiRNAs (71). These studies show that miRNA exhibits high affinity for exosomes, but further research is still needed with respect to the mechanistic interaction between exosomes and specific miRNAs, and on whether all exosomes from one cell type have a similar distribution of specific miRNAs.

## Exosomal miRNAs As Biomarkers in Disease Diagnosis

The clinical diagnosis of IS currently and mainly depends upon MRI and computed tomography; however, some patients cannot complete these examinations due to the presence of metal stents or steel plates implanted in the body. When the symptoms are mild or in the initial stage of the disease, it is very difficult for doctors to distinguish stroke from other CNS diseases, and to discriminate between ischemic stroke and hemorrhagic stroke based on imaging results. Although a growing number of biomarkers such as those involved in inflammatory reactions, apoptosis, and oxidative damage have been uncovered, these markers have certain limitations in clinical application due to the problems of detection methods and their specificity and sensitivity. Therefore, finding an effective and reliable biomarker for IS remains a great challenge. Fortuitously, it has been shown that circulating exosomes and exosomal miRNAs are closely related to ischemic cerebrovascular disease (77, 78). Compared with cellular or free miRNAs, exosomal miRNAs carry higher sensitivity and specificity, and they are considered to be better biomarkers of stroke—especially in the ultra-early and early stages when diagnosis is more difficult. The potential clinical value of exosomes can be attributed to their surface markers and molecular cargos (16, 17), relatively low immunogenicity, relatively long half-life in the circulation, their ability to cross the BBB (14, 15, 79), and their potential roles as mediators of regenerative responses. Therefore, miRNAs from exosomes are more stable and accurate for use in the determination of biomarkers related to ischemic stroke (Table 1).

**Table 1:** Exosomal miRNAs as biomarkers for stroke diagnosis.

**Exosomal Source**	**Exosomal Contents**	**Clinical application**	**Stage of IS**	**Area under the curve (AUC)**	**References**
human serum	exosomal miR-223	identifying AIS, predicting stroke severity, and short-term outcomes	acute ischemic stroke (AIS)	AUC was 0.859.	(38)
human serum	Exosomal miR-9 and miR-124	identifying AIS, predicting stroke severity	acute ischemic stroke (AIS)	AUC of exosomal miR-124 was 0.6976 and exosomal miR-9 was 0.8026	(72)
human serum	exosomal miR-134	identifying AIS, predicting stroke severity	acute ischemic stroke (AIS)	AUC was 0.834	(73)
human serum	exosomal miR-152-3p	identifying AIS,	acute ischemic stroke (AIS)	AUC was 0.935	(74)
human plasma	exosomal miR-21-5p and miR-30a-5p	distinguishment among AIS, HIS, SIS, and RIS,	hyperacute phase of IS (HIS)	The AUCs for miR-21-5p were 0.714 in the SIS group and 0.734 in the RIS group. The AUCs for miR-30a-5p were 0.826 in the HIS group and 0.438 in the AIS group.	(75)
human plasma	exosomal miR-422a and miR-125b-2-3p	identifying acute and subacute phases, with the latter exhibiting higher diagnostic value	subacute Phase of IS(SIS)	AUC values for miR-422a and miR-125b-2-3p in the subacute phase were 0.971 and 0.889; miR-422a in the acute phase was 0.769.	(76)

### Acute Phase of IS

The levels of exosomal miR-223 in patients with acute ischemic stroke (AIS) were significantly increased and positively correlated with NIHSS score. Furthermore, the expression of miR-223 in patients with a poor prognosis was elevated relative to the expression in patients with a favorable prognosis. We evaluated the diagnostic value of miR-223 using receiver operating characteristic (ROC) curve analysis, and found that the area under the curve (AUC) for miR-223 exhibited high sensitivity and specificity in the diagnosis of stroke (38). The increase in exosomal miR-223 was also associated with IS occurrence, stroke severity, and short-term prognosis. However, further studies with larger sample sizes are needed to assess the clinical application of exosomal miR-223 as a new biomarker for the diagnosis of IS. Investigators revealed that the expression levels of exosomal miR-9 and miR-124 in the serum of patients with AIS were markedly elevated, with their AUCs at 0.6976 and 0.8026, respectively; and that they correlated with the severity of stroke (72). Blood samples from patients with AIS and from healthy control subjects were collected to isolate exosomes and analyze exosomal miR-134 expression by RT-qPCR, and our results showed that miR-134 in exosomes were markedly augmented within 24 h after stroke onset. The AUC of miR-134 was 0.834 (95% confidence interval, 0.88–0.97); and the level of secreted miR-134 was correlated with the National Institutes of Health Stroke Scale (NIHSS) score and infarct volume, and positively correlated with serum interleukin-6 (IL-6), high sensitivity C-reactive protein (hsCRP), and a poor prognosis for stroke. These data thus suggest that exosomal miR-134 may constitute a novel biomarker for the diagnosis and prognosis of stroke (73). Research has shown that serum exosomal miR-152-3p levels in patients with AIS were significantly attenuated relative to those of healthy subjects, and that the reduced expression of exosomal miR-152-3p was correlated with the severity of the neurologic deficit. In addition, the exosomal miR-152-3p level was lowest in large-artery atherosclerosis (LAA) patients, and its expression diminished in the acute phase relative to the recovery phase. The AUC of the exosomal miR-152-3p level was 0.935, which indicated that exosomal miR-152-3p may be a potential diagnostic marker for AIS (74).

### Hyperacute Phase of IS

The above studies have highlighted the diagnostic value of plasma-exosome miRNAs in AIS as well as showing their correlation with disease severity. Here we discuss the potential predictive value of plasma exosomal miRNAs in different stages of stroke. One study (75) revealed plasma exosomal miR-30a-5p and miR-21-5p levels in the four stages of IS. A total of 143 patients with IS were divided into the following four groups: the hyperacute phase of IS (HIS, within 6 h); acute phase of IS (AIS, days 1–3 and days 4–7); subacute phase of IS (SIS, days 8–14); and the recovery phase of IS (RIS, days > 14). An additional 24 non-stroke patients were included as controls. The plasma exosomal miR-21-5p level appeared to be enhanced in the RIS and SIS groups; the miR-30a-5p level in the HIS group was elevated, and that in the AIS group was significantly reduced. In the AIS group, both miRNAs were decreased compared with those in the HIS group. The AUCs of miR-21-5p were 0.714 in the SIS group and 0.734 in the RIS group, and the AUCs of miR-30a-5p were 0.826 in the HIS group and 0.438 in the AIS group. These findings suggest that plasma miRNA-30a-5p and miR-21-5p may be valuable biomarkers in the diagnosis of IS as well as in distinguishing among the four stages of IS—particularly miRNA-30a-5p in the diagnosis of the HIS phase. The diagnosis of the hyperacute stage of IS is therefore very important in guiding clinical treatment.

### Subacute Phase of IS

A previous study (76) showed that plasma levels of exosomal miR-422a and miR-125b-2-3p in the subacute phase were lower than those in the acute phase, and that the levels of miR-422a were higher in the acute phase. ROC analysis revealed that the AUC values for exosomal miR-125b-2-3p and miR-422a in the subacute phase were also higher than those in the healthy control group. Plasma exosomal mir-125b-2-3p and mir-422a may therefore be considered blood biomarkers for the diagnosis of IS, and can be used to identify acute and subacute phases, where the latter exhibits higher diagnostic value (75).

Collectively, these studies indicate that exosomal miRNAs serve potential applications as biomarkers in the diagnosis of stroke. However, there are still some limitations to the clinical application of exosomal miRNA. For example, the methods used to extract exosomes are inconsistent, as are the identification of exosomes and the methods used to detect exosomal miRNAs. The entire operational process is thus relatively complex and there is no unified methodology. The clinical diagnostic application of exosomal miRNAs is therefore still unclear at this time. Additionally, the differential expression patterns of exosomal miRNAs at different stages of IS suggest that they may be useful as a diagnostic biomarker of each IS phase. There is currently a lack of comparability for exosomal miRNAs in patient and animal plasma and CSF by high-throughput sequencing, as well as a lack of further validation of the screened exosomal miRNAs. We look forward to high-throughput screening of ectomiRNAs in hyperacute, acute, subacute, and convalescent stages of stroke to obtain circulating exosomal miRNAs of diagnostic value. In addition, values of circulating exosomal miRNAs used to predict the presence of IS and their relationships to the severity of neurologic deficits associated with IS and subsequent stroke risk after IS require further analysis.

## Exosomal miRNAs as Therapeutic Agents in the Treatment of Stroke

In addition to drug treatment of stroke, animal experiments have shown that intravenous injection of pluripotent stem cells can enhance the recovery of neurologic function after stroke (80). Although stem cells exert therapeutic effects on IS (81), the migration of stem cells to the cerebral ischemic area after intravenous injection remains unclear. In addition, how to deliver drugs safely and effectively still requires solutions in IS treatment. Researchers have recently found a novel mechanism by which to explain the role of mesenchymal stem cells (MSCs) in the recovery of neural function. MSCs can release exosomes that can reach brain tissue, act on parenchymal cells, regulate gene expression and protein production of nerve cells, and also accelerate nerve regeneration and functional recovery (82). Because exosomes can cross the BBB, stem cells may play a protective role in cerebral ischemia through the secretion of exosomes. There is now growing interest in the use of stem cell-derived exosomes in the field of IS. In addition, exosomes exhibit other unique and advantageous properties in the context of drug delivery, including their low immunogenicity, innate stability, and high-delivery efficiency. Exosomes may therefore provide a targeting delivery system within ischemic brains (83). Exosomes are released by endothelial cells in femoral arteries, and they mediate the protective effects of remote ischemic postconditioning (RIP) on neurologic damage (84). Exosomes possess broadly applicative prospects in the treatment of cerebral ischemia. In one study, magnetic nanovesicles (MNVs) derived from iron oxide nanoparticle (IONP)-harboring MSCs markedly improved ischemic-lesion targeting and therapeutic outcomes (85). Intravenous injection of urinary stem cell exosomes (USC-Exos) advances neurogenesis and reduce neurologic deficits in IS rats. *In vitro*, USC-Exos promote the proliferation and differentiation of neural stem cells after oxygen glucose deprivation/re-oxygenation (OGD/R). The neurogenic effect of USC-Exos may be related to exosomal microRNA-26a (miR-26a) (86) (Table 2).

**Table 2:** Exosomal miRNAs as therapeutic agents for ischemic stroke.

**Exosomes Source**	**Exosomes Contents**	**Therapeutic effect**	**References**
USCs	miR-26a	neurogenesis	(86)
MSCs	miR-133b	neural plasticity and functional recovery	(87)
microglia	miR-137	reduce the infarct volume and behavioral defects	(88)
BMSCs	miR-138-5p	reducing neurological impairment by promoting proliferation and inhibiting inflammatory responses of astrocytes	(89)
MSCs	miR-17-92	increasing neural plasticity and functional recovery after stroke	(90)
circulating EPCs/endothelial cells	miR-126	inducing neurorestorative effects	(91, 92)
modified exosomes with rabies virus glycoprotein (RVG)	miR-124	promoting cortical neural progenitors to obtain neuronal identity and protecting against ischemic injury by robust cortical neurogenesis.	(93)
M2 microglial	miR-124	reducing formation of glial scar, improving the recovery	(94)
brain endothelial cell	miR-126-3p	increases neurite outgrowth	(95)
plasma	miR-451	increased the survival rate of Neuro-2a cells	(96)

*USCs, urinary stem cells; MSCs, multipotent mesenchymal stromal cells; BMSCs, bone marrow stromal cells*.

Exosomal miRNAs are significant in the exosome-based treatment of stroke. The inherent ability for neurogenesis after stroke is very poor, making it is difficult for the nervous system to repair itself. Targeted exosome miRNAs can favor neurogenesis, which would be very helpful in enhancing the prognosis of patients with stroke. Therefore, we will primarily explore the role of exosomal miRNAs in the treatment of stroke. *In vitro*, OGD astrocytes treated with Ex-miR-133b+ notably promoted neurite branching and lengthening of embryonic cortical neurons, and exosomes obtained by miR-133b+ multipotent MSCs increased neural plasticity and functional recovery. *In vivo*, Ex-miR-133b+ treatment significantly increased the functional improvement of ischemic border areas and neurite remodeling/brain plasticity (87). The exosomal miR-137 of microglia reduced infarct volume and behavioral defects in ischemic mice (88). Exosomal miR-138-5p derived from bone marrow stromal cells (BMSCs) alleviated neural lesions by promoting the proliferation of astrocytes, inhibiting apoptosis, and regulating inflammatory factors by downregulating LCN2 (89). Exosomes rich in miR-17-92 clusters enhance neural plasticity and functional recovery in middle cerebral artery occlusion(MCAO) rats (87), and miR-126+ exosomes can promote regeneration of the facial nerve in mice with type 2 diabetes mellitus and stroke (92).

It has been reported that exosomes modified by rabies virus glycoprotein (RVG) fused with exosomal protein lysosome-associated membrane glycoprotein 2B (LAMP2b) further enhanced the targeting of exosomes, and that miR-124 was effectively delivered to the infarct site. These exosomes promoted the acquisition by cortical neural progenitor cells of neuronal characteristics, augmented cortical neurogenesis, and reduced ischemic injury (93). Recent research showed that exosomal miR-124 from M2 microglia reduced glial scar formation, bolstered recovery after stroke, and the migration and proliferation of astrocytes were alleviated by inhibiting the expression of signal transducer and activator of transcription 3 (STAT3) (94). Brain endothelial cell-derived exosomal miR-126-3p altered brain plasticity, promoting neurite growth and inhibiting PC12 cell injury and apoptosis (95).

Remote ischemic preconditioning (RIPC) comprises an effective neuroprotective protocol. The blood exosomes after RIPC transmit signals throughout the body. Infusion of RIPC-concentrated plasma also significantly reduced infarct size in mice with cerebral ischemia via the HIF-1α-induced signaling pathway (97). Exosomal miR-451a occupies a significant role during cerebral ischemic preconditioning (cerebral IPC), reducing cerebral ischemia-reperfusion injury; and it can increase the survival rate of Neuro-2a cells and activate Rac1 and its downstream pathway, thus reducing reperfusion injury (96). The neuroprotective effect of exosomes and exosomal miRNAs from plasma after ischemic preconditioning, however, remains to be analyzed.

At present, research on the therapeutic effects of exosomal miRNAs in IS has been limited to animal experiments and *in vitro* experiments, and further translational research is needed before such findings can be used to initiate clinical trials in humans.

## Mechanism Underlying the Treatment of IS

### Exosomal miRNA Involvement in the Pathogenesis of Cerebral Ischemia

In the state of cerebral ischemia/hypoxia, exosomal miRNAs secreted by nerve cells and endothelial cells participate in brain tissue injury. Through next-generation sequencing, significant differences have been uncovered in the expression levels of 45 secretory miRNAs between exosomes collected from normoxic culture medium and oxygen- and glucose-deprived/reoxygenated medium. Bioinformatics analysis showed that this phenomenon involves many pathways, including cell survival and death and neuronal signal transduction. Furthermore, the exosomes of cortical neurons from OGD/R-treated, cultured cortical neurons significantly damaged their viability, diminished primary or total neurite number, and inhibited the growth of neurites (98). Studies have revealed that the intestinal epithelial cell line-6 (IEC-6) and cortical neurons cultured *in vitro* secreted exosomal miRNAs, and that OGD-treated IEC-6 cells increased exosomal miRNA levels. OGD-IEC-6 co-cultured neurons also secreted elevated levels of exosomal miRNAs related to apoptosis, necroptosis, and/or focal death events. Thus, I/R-injured intestinal epithelial cells can induce cortical neuron death by releasing exosomal miRNAs (99).

In another study, exosomes were obtained from microglia and their miRNA expression was determined, and the research shows that exosomal miR-424-5p was one of the most differentially expressed miRNAs. *In vitro*, the intervention by microglial exosomes treated with OGD on cerebral microvascular endothelial cells (BMECs) created significant cellular damage and increased permeability. After inhibiting miR-424-5p, the damaging effect was significantly ameliorated.

Overexpression of mir-424-5p can notably aggravate BMEC injury and permeability caused by OGD. *In vivo*, inhibiting mir-424-5p can reverse nerve injury *in vivo*. Through bioinformatics analysis, mir-424-5p acted on STAT3 signaling pathway mediated by FGF2 (100). Exosomal miR-424-5p can therefore cause nerve injury and endothelial cell destruction, and the mechanism may be related to the FGF2-mediated STAT3-signal-transduction pathway.

The aforementioned studies show that dozens of exosomal miRNAs are involved in the onset of cerebral ischemic injury. It is therefore critical to ascertain the most meaningful exosomal miRNAs and further explore their crucial roles and regulatory mechanisms in the occurrence and development of cerebral infarction.

### Mechanism Underlying Brain Protection in IS

**Figure 2:**
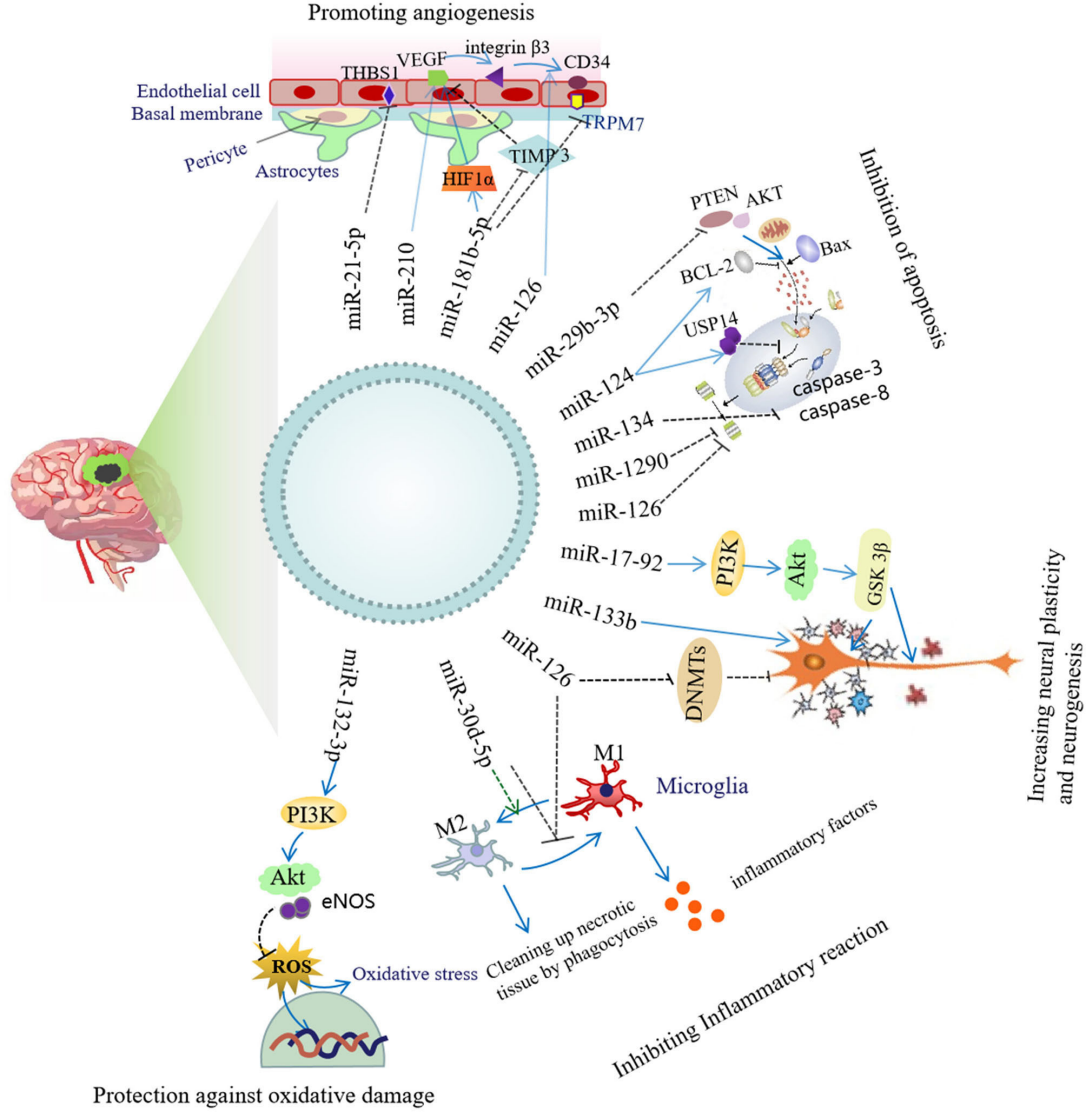
Neuroprotective mechanism of exosomal miRNA.

Exosomes secreted by stem cells have been reported to act as carriers responsible for miRNA transfer to lesion sites in stroke (17). In this manner, exosomes enable stem cells to accomplish their regulatory roles in functional recovery after stroke by delivering specific miRNAs that can provide therapeutic modifications to recipient cells. Exosomes secreted by stem cells and various neural cells carry a variety of miRNAs that are integral to improving neural function after cerebral ischemia, and their related mechanisms of action involve inhibiting inflammatory responses and apoptosis, as well as improving neural repair and plasticity. ADSCs-Exos promote angiogenesis and the mobility of brain microvascular endothelial cells after OGD (97). Exosomes portray regulatory roles by delivering pre-wrapped cargos that contain miRNAs, lncRNAs, and proteins to recipient cells (101, 102). Exosomal miRNAs from a variety of cell types show encouraging neural-regenerative capabilities, including improving neural function and neurite outgrowth; inducting angiogenesis, anti-apoptosis, and anti-inflammation; and promoting neural plasticity and neurogenesis following stroke (Figure 2).

### Inhibition of Inflammatory Responses

Systemic administration of miR-126+ exosomes derived from ADSCs significantly enhanced the levels of von Willebrand factor (an endothelial cell marker) and doublecortin (a neuroblastoid marker), reduced neuronal death, increased cellular proliferation, and implemented the functional recovery of rats with stroke as compared with miR-126- exosome therapy. In addition, miR-126+ exosomes inhibited the activation of microglia and the expression of inflammatory factors *in vitro* and *in vivo* (103). It has been shown in IS patients and animal models that the levels of inflammatory cytokines increased, while the levels of anti-inflammatory cytokines (i.e., IL-4, IL-10) and miR-30d-5p were attenuated. *In vitro*, the inhibition of autophagy significantly reduced the inflammatory response induced by OGD. And, *in vivo*, miR-30d-5p+ exosomes derived from ADSC significantly reduce the brain-injury area of cerebral infarction and prevented brain damage by suppressing autophagy-mediated polarization of microglia to the M1 phenotype (104).

### Inhibition of Apoptosis

Exosomes derived from M2 microglia reduce neuronal apoptosis, infarct size, and behavioral impairments after OGD, and this is achieved by miR-124 and its downstream target USP14; and MiR-124-M2 microglia-derived exosomes reversed this neuroprotective effect (65). Exosomes derived from BMSCs inhibited oligodendrocyte apoptosis by exosomal miR-134, which then negatively regulated the caspase-8-dependent apoptotic pathway (105). Exosomal delivery of miR-29b-3p suppressed apoptosis in MCAO rats and OGD cells by upregulating the expression of Bcl-2 and reducing the expression of Bax and cleaved caspase 3. Other studies have shown that the antiapoptotic effect of exosomal delivery of miR-29b-3p was achieved through the regulation of the PTEN/Akt-signaling pathway, participating significantly in mitigating hypoxic-ischemic brain damage (106). Exosomes derived from endothelial progenitor cells (EPC-EXs) containing miR-126 have also been shown to inhibit apoptosis, and exosomal miR-126 levels correlated negatively with infarct volume and apoptosis, and positively with microvessel density. In addition, an miR-126 inhibitor reversed the protective effect of EPC-EXs on hypoxia-induced neuronal apoptosis and axon growth, and use of a PI3K inhibitor also showed such an inhibitory effect (91). Investigators have shown that the expression of Cav-1 in neurons was upregulated after ischemia, increasing the intake of EVs; and that EV protected neurons by inhibiting apoptosis via miR-1290 (107).

### Promotion of Angiogenesis

RGD exo: miR-210 injected intravenously into an ischemic brain-injured area using a transient MCAO mouse model resulted in an increase in mir-210; and the expression levels of integrin β3, VEGF, and CD34 were significantly upregulated, with mouse survival rate also enhanced (108).

Exosomes secreted from HUVECs and EPCs manifest similar morphologies, size distributions, and biologic characteristics/activities, but those derived from EPCs are relatively more abundant. Therefore, EPCs may be a robust source of exosomes that can be used to promote vascular repair (109). Exosomes secreted from EPCs promote re-endothelialization, augment the proliferation and migration of endothelial cells, and enhance the levels of angiogenesis-related factors (110). Another group also concluded that EPC exosomes promoted re-endothelialization; and that miR-21-5p was highly expressed in EPC exosomes, and specifically inhibited the expression of thrombospondin-1, an angiogenesis inhibitor (111).

One study disclosed that exosomes from ADSCs (ADSCs Exos) stimulated the mobility and angiogenesis of OGD brain microvascular endothelial cells (BMECs). This effect was related to exosomal miR-181b-5p, which also increased the migration distance and tube length of BMECs. Transient receptor potential melastatin 7 (TRPM7) is a downstream target of mir-181b-5p, and its levels were reduced in BMECs cultured with exosomal miR-181b-5p, but not in those cultured with exosomal miR-212. In addition, exosomal miR-181b-5p elevated HIF-1α and VEGF expression levels, whereas the expression of tissue inhibitor of metalloproteinase 3 (TIMP-3) diminished. Overexpression of TRPM7 also attenuated the regulation of exosomal miR-181b on angiogenesis-related factors and on BMEC migration and tube formation (101).

### Protective Capability Against Oxidative Damage

MSC exosomes and miR-132-3p-overexpressing MSC exosomes have been produced by mouse MSCs transfected with scrambled control or miR-132-3p mimics in order to analyze the effects of exosomal miR-132-3p on endothelial cells injured by hypoxia/reoxygenation (H/R). The results showed that MSC-Exs transferred miR-132-3p into endothelial cells, which then downregulated the target RASA1 and upregulated Ras expression and the phosphorylation of downstream PI3K. MSC-Exs miR-132-3p more significantly suppressed apoptosis, ROS production, and tight-junction damage of endothelial cells compared with MSC Exs, both *in vitro* and *in vivo*. These effects were related to the increase in phosphorylated Akt and eNOS levels, which were abrogated by PI3K inhibitor (LY294002) or Ras inhibitor (nsc23766) (112).

### Augmentation of Neural Plasticity and Neurogenesis

In one study, the effect of miR-17-92+ exosomes obtained from MSCs was evaluated on neurologic recovery, and it was demonstrated that these exosomes improved neurologic function; and enhanced neurogenesis, oligodendrogenesis, and neurite remodeling/neuronal dendritic plasticity in the ischemic boundary zone (IBZ) post-MCAO. These effects of MSC exosomal miR-17-92 were achieved by targeting phosphatase and tensin homologs, and activating the mechanistic targets involved in PI3K, protein kinase B, rapamycin, and glycogen synthase kinase 3β (90).

Scientists have previously examined whether exosomes from miR-133b-overexpressing MSCs play a therapeutic role in amplification. After treatment with miR-133b (+) MSCs for 14 days, the levels of miR-133b in exosomes collected from the cerebrospinal fluid of MCAO rats significantly increased, and the expression of connective tissue growth factor and RAS homologous gene family member A significantly decreased in the IBZ (113). After MCAO, exosomes from miR-133b-overexpressing MSCs (Ex-miR-133b+) treated for 28 days significantly improved neurologic functional recovery, increased brain exosomes, and increased neurite remodeling/brain plasticity in the IBZ (87). *In vitro*, Ex-miR-133b+ augmented neurite branching and elongation of cultured cortical neurons. Therefore, the exosomes obtained from MSCs overexpressing miR-133b can improve the neural plasticity and functional recovery after stroke by stimulating astrocytes to promote the secondary release of exosomes (87). Furthermore, it has been shown that MSCs communicate with brain parenchymal cells and may regulate neurite outgrowth by transferring miR-133b to neural cells via exosomes. Cel-mir-67 in MSCs was transferred to astrocytes via exosomes with a diameter of 50–100 nm, and a gap-junction intercellular communication inhibitor prevented exosomal miRNA communication by inhibiting exosomal release (114).

Transient limb ischemia can induce cerebral ischemic tolerance and RIPC. Interestingly, exosomal miR126 from RIPC serum exhibited a neuroprotective effect on SH-SY5Y neuronal cells, and the mechanism of effect was related to the downregulated expression of DNA methyltransferase (DNMT) 3B, the target gene of miRNA-126 (115) (Table 3).

**Table 3:** Mechanism underlying the actions of exosomal miRNAs from different cells on ischemic stroke.

**Exosomes Source**	**Exosomes Contents**	**Mechanisms underlying IS**	**References**
**Inhibiting inflammatory response**			
ADSCs	miR-126	Inhibiting microglial activation and the expression of inflammatory factors	(103)
ADSCs	miR-30d-5p	Suppressing autophagy and promoting M2 microglia/macrophage polarization	(104)
**Inhibition of apoptosis**			
M2 microglia	miR-124	Inhibiting apoptosis by downstream target USP14	(65)
BMSCs	miR-134	Suppressing OLs apoptosis by negatively regulating the caspase-8-dependent apoptosis pathway	(105)
BMSCs	miR-29b-3p	Decreasing the expression of Bax and cleaved caspase 3 and upregulated Bcl-2, negative regulation of PTEN and activation of Akt	(106)
EPC	miR-126	Inhibiting apoptosis and enhancing axon growth	(91)
HUVECs	miR-1290	Attenuating apoptosis	(84, 107)
**Promoting angiogenesis and repairing the BBB**			
RGD-exo	miR-210	Increasing integrin β3, vascular endothelial growth factor (VEGF) and CD34	(108)
EPC	miR-21-5p	Suppressing the expression of an angiogenesis inhibitor THBS1 in the recipient EC.	(111)
ADSC-Exos	miR-181b-5p	Decreasing TRPM7 mRNA and protein levels, upregulating the protein expression of HIF1α and VEGF, and downregulated the protein expression of TIMP3	(101)
circulating EPCs	miR-126	Promoting angiogenesis, increasing axon density, myelin density, vascular density, arterial diameter	(92)
**Protection against oxidative damage**			
MSCs	miR-132-3p	Decreasing ROS production, downregulating the target protein RASA1, upregulating the expression of Ras and the downstream PI3K phosphorylation, those were associated with increased levels of phosphorylated Akt and eNOS.	(112)
Increasing neural plasticity and neurogenesis
MSCs	miR-17-92	Improvement of neurological function and enhancements of oligodendrogenesis, neurogenesis, and neurite remodeling/neuronal dendrite plasticity by inhibiting phosphatase and tensin homolog and increasing the phosphorylation of phosphatase and tensin homolog downstream proteins, PKB, mechanistic target of rapamycin, and GSK3β	(90)
MSCs	miR-133b	Increasing axonal plasticity and neurite remodeling; regulating neurite outgrowth	(87, 113, 114)
RIPC serum	miR-126	Neuroprotective effects by downregulating the expression of DNMTs in neural cells	(115)

*ADSCs, Adipose-derived stem cell; EPC, Endothelial progenitor cells; HUVECs, Human vascular endothelial cells; MSCs, Multipotent mesenchymal stromal cells; RIPC, Remote ischemic preconditioning; TIMP3, tissue inhibitor of metalloproteinase 3; THBS1, Thrombospondin-1; GSK3β, glycogen synthase kinase 3β; PKB, protein kinase B; DNMT, DNA methyltransferase*.

Herein we discussed the mechanisms underlying actions of exosomal miRNAs in the treatment of cerebral ischemia, which involves inhibiting inflammatory responses and apoptosis; promoting angiogenesis; protecting the BBB; combating antioxidant damage; and encouraging nerve repair and plasticity. Further studies on the governing mechanisms of exosomal miRNAs will assist us in understanding their physiologic and pathologic functions. As current research is primarily focused on exosomal miRNAs secreted by pluripotent MSCs, further research is required to unravel the types of exosomes released from different cell sources and those that are most effective. We also need to understand whether the mechanism of exosomal miRNA action involves autophagy, calcium overload, or cell death; and to appreciate their regulation of related pathways. Exosomal miRNAs constitute a novel therapeutic strategy and may augur great benefit in stroke treatment. In-depth study of the mechanisms subserving exosomal action regarding IS will provide additional evidence for the clinical localization and therapeutic effects of exosomal miRNAs.

## Conclusions

Ischemic stroke prevention and treatment are common concerns worldwide. Reasonable prevention, early diagnosis, and effective treatment are thus important strategies needed to improve the prognosis of IS patients. Exosomes exhibit potential clinical value as both diagnostic biomarkers and therapeutic drug carriers in stroke. Moreover, since exosomes released by brain cells manifest low immunogenicity, they can cross the BBB and maintain a long half-life in the systemic circulation; it is therefore possible to isolate and analyze the molecules contained in these circulating exosomes. This information may identify efficient biomarkers and more efficient monitoring of pathologic progress in CNS diseases, as well as be used to assess any effects of treatment. However, there are challenges that exist with respect to this approach. Future work will necessitate large-scale population studies, and the specificities and sensitivities of exosomal candidates need to be confirmed in further clinical investigations. We posit that a single type of exosomal miRNA will not be able to achieve the purpose of a diagnostic criterion; rather, a combination of multiple exosomal miRNAs may show greater feasibility as a collective diagnostic marker for stroke, and allow the differentiation between stroke and TIA. Therefore, we wish to compare exosomal miRNAs in patient plasma, animal plasma, and cerebrospinal fluid using high-throughput sequencing; and further verify the screened exosomal miRNAs. We also anticipate high-throughput screening of a group of exosomal miRNAs rather than a single one in the hyperacute, acute, subacute, and convalescent stages of stroke so as to obtain circulating exosomal miRNAs with diagnostic value.

In the present review, we presented and scrutinized possible applications of diverse stem cell-derived exosomes in stroke. Collectively, these findings suggest that diversified stem cell-derived exosomes possess therapeutic potential in the treatment of neurological diseases by inhibiting apoptosis and promoting both angiogenesis and neural repair in preclinical studies and may serve as biomarkers in both preclinical and clinical studies. However, the analysis of exosomes is still in its nascency, and many problems exist that require clarification, such as whether there is a connection between the exosomes released by ischemic tissue and the exosomes released by distal organs, whether exosomal release is consistent, and whether there are differences in quantity and content after cerebral ischemia. Whether the secreted miRNA released by the same infarct site but in different patients is also the same needs to be further resolved. The investigation of exosomal miRNAs from different cells for the treatment of stroke has been limited at present to basic research in model systems and is still far from demonstrating clinical application. Future studies are warranted to further identify the optimal cell-produced exosomes that are suitable for the diagnosis and treatment of stroke. In addition, considerable future work is essential to screening for the most effective exosomal miRNAs and to delineate their roles in the development of stroke.

## Author Contributions

YX: conceptualization, writing-original draft preparation, writing-reviewing, and editing. YG: conceptualization, investigation, and funding acquisition. YH: writing-review and editing. SX: conceptualization. FL: collection and collation of data. All authors read and approved this manuscript for publication.

## Funding

This work was supported by the National Key Technologies and Program (2018YFC1705000) and the Postdoctoral Research Foundation of China (2020M680470).

## Conflict of Interest

The authors declare that the research was conducted in the absence of any commercial or financial relationships that could be construed as a potential conflict of interest.

## Publisher's Note

All claims expressed in this article are solely those of the authors and do not necessarily represent those of their affiliated organizations, or those of the publisher, the editors and the reviewers. Any product that may be evaluated in this article, or claim that may be made by its manufacturer, is not guaranteed or endorsed by the publisher.
